# Platelets interact with CD169^+^ macrophages and cDC1 and enhance liposome-induced CD8^+^ T cell responses

**DOI:** 10.3389/fimmu.2023.1290272

**Published:** 2023-11-20

**Authors:** Joanna Grabowska, Valentine Léopold, Katarzyna Olesek, Maarten K. Nijen Twilhaar, Alsya J. Affandi, Mieke C. Brouwer, Ilse Jongerius, Admar Verschoor, Cees van Kooten, Yvette van Kooyk, Gert Storm, Cornelis van ‘t Veer, Joke M. M. den Haan

**Affiliations:** ^1^ Department of Molecular Cell Biology and Immunology, Amsterdam UMC location Vrije Universiteit Amsterdam, Amsterdam, Netherlands; ^2^ Cancer Biology and Immunology Program, Cancer Center Amsterdam, Amsterdam, Netherlands; ^3^ Cancer Immunology Program, Amsterdam Institute for Infection and Immunity, Amsterdam, Netherlands; ^4^ Center of Experimental and Molecular Medicine, Amsterdam UMC, University of Amsterdam, Amsterdam, Netherlands; ^5^ Department of Anesthesiology and Critical Care, Paris University, Lariboisière Hospital, Paris, France; ^6^ Inserm UMR-S 942, Cardiovascular Markers in Stress Conditions (MASCOT), University of Paris, Paris, France; ^7^ Department of Immunopathology, Sanquin Research and Landsteiner Laboratory, Amsterdam University Medical Centre, Amsterdam Infection and Immunity Institute, Amsterdam, Netherlands; ^8^ Department of Dermatology, University of Lübeck, Lübeck, Germany; ^9^ Department of Otorhinolaryngology, Technische Universität München and Klinikum Rechts der Isar, Munich, Germany; ^10^ Department of Medicine, Division of Nephrology and Transplant Medicine, Leiden University Medical Center, Leiden, Netherlands; ^11^ Department of Pharmaceutics, Faculty of Science, Utrecht University, Utrecht, Netherlands; ^12^ Department of Biomaterials, Science and Technology, Faculty of Science and Technology, University of Twente, Enschede, Netherlands; ^13^ Department of Surgery, Yong Loo Lin School of Medicine, National University of Singapore, Singapore, Singapore

**Keywords:** platelets, liposomes, vaccination, CD169 macrophage, cDC1, T cells, ganglioside GM3

## Abstract

Historically platelets are mostly known for their crucial contribution to hemostasis, but there is growing understanding of their role in inflammation and immunity. The immunomodulatory role of platelets entails interaction with pathogens, but also with immune cells including macrophages and dendritic cells (DCs), to activate adaptive immune responses. In our previous work, we have demonstrated that splenic CD169^+^ macrophages scavenge liposomes and collaborate with conventional type 1 DCs (cDC1) to induce expansion of CD8^+^ T cells. Here, we show that platelets associate with liposomes and bind to DNGR-1/Clec9a and CD169/Siglec-1 receptors *in vitro*. In addition, platelets interacted with splenic CD169^+^ macrophages and cDC1 and further increased liposome internalization by cDC1. Most importantly, platelet depletion prior to liposomal immunization resulted in significantly diminished antigen-specific CD8^+^ T cell responses, but not germinal center B cell responses. Previously, complement C3 was shown to be essential for platelet-mediated CD8^+^ T cell activation during bacterial infection. However, after liposomal vaccination CD8^+^ T cell priming was not dependent on complement C3. While DCs from platelet-deficient mice exhibited unaltered maturation status, they did express lower levels of CCR7. In addition, in the absence of platelets, CCL5 plasma levels were significantly reduced. Overall, our findings demonstrate that platelets engage in a cross-talk with CD169^+^ macrophages and cDC1 and emphasize the importance of platelets in induction of CD8^+^ T cell responses in the context of liposomal vaccination.

## 1 Introduction

Platelets are small (2 to 4 µm in size), anucleated blood components that are essential for coagulation. Although platelets have a rather short life-span of 7-10 days, they are abundantly present in the circulation (150-400 x 10^9^/liter of blood in humans). To fulfill their role, platelets are produced daily in high numbers (approximately 100 billion) by bone marrow megakaryocytes in a process called thrombopoiesis (1). Platelets are equipped with a variety of biologically active molecules that are packaged into three types of secretory granules: α-granules, dense granules and lysosomes. Alpha-granules are the most numerous and of high relevance for immune responses, as they contain adhesion molecules e.g. CD62P, platelet microbicidal proteins and microbicidal chemokines e.g. CCL5, amongst many (2). Upon activation, platelets undergo cytoskeleton rearrangements and release or translocate granule-stored cargo to the surface. Activated platelets also produce microparticles via cytoplasmic blebbing, which is another form of platelet communication with their surroundings (3).

Apart from playing a fundamental role in hemostasis, platelets are being increasingly appreciated as immune modulators. This can be attributed to their ample presence in the circulation and biological characteristics that include direct anti-bacterial effects, interaction with leukocytes and activation of immune responses. Owing to their expression of pathogen recognition receptors (PRRs) such as Toll-like receptors (TLRs), platelets have the capacity to sense pathogen-associated-molecular-patterns (PAMPs) and danger-associated-molecular-patterns (DAMPs) and thus have been described as immune sentinels. Platelets have been shown to interact with pathogens such as bacteria and viruses by binding, engulfment or direct killing via secretion of antimicrobial factors, which often leads to platelet activation and degranulation (4, 5). Engulfment of bacteria by platelets was demonstrated to prevent bacterial dissemination and promote bacterial clearance (6, 7). Similarly, platelet internalization of viruses such as influenza, HIV and Dengue results in removal of the viral particles as well as platelets (8–11).

Platelets contribute to pathogen control not only by directly interacting with invading bacteria and viruses but also by engaging other immune cells. Accordingly, CD62P-PSGL-1 and CD40-CD40L axes are both exploited by platelets to associate with monocytes and neutrophils (2, 12). In addition, neutrophil-platelet aggregation that leads to polymorphonuclear neutrophils (PMN) activation and NETosis occurs in a TLR-mediated manner (13). Next to surface-expressed receptors, platelet-secreted factors such as CCL5 and CXCL4, also mediate platelet-leukocyte interaction by attracting monocytes (14). Finally, platelets engage macrophages and enhance their phagocytosis via GPIb-CD11b (15) as well as collaborate with liver macrophages also known as Kupffer cells in destroying invading bacteria via GPIb-vWF (16).

Apart from having an impact on monocytes, neutrophils and macrophages, platelets play a role in activation of adaptive immune responses. Various studies report platelet-DC cross-talk and its effects on DC recruitment, differentiation, activation and maturation (17–19). In addition, platelets can provide ‘help’ by interacting with CD40 on DC via surface-expressed or secreted CD40L underscoring the importance of CD40L-CD40 axis for subsequent priming of T and B cells (20–26). Using an adenoviral infection model it was shown that only activated platelets from WT mice, and not from CD154KO mice, promoted DC maturation, B cell class switching and enhanced primary and secondary anti-viral cytotoxic T lymphocyte (CTL) responses (17). Similarly, expansion of anti-Lymphocytic choriomeningitis virus (LCMV) CD8^+^ T cells was also demonstrated to be partially mediated by platelet-derived CD40L (26). Interestingly, platelets have been also described to shuttle *Listeria monocytogenes* to cross-presenting DCs in a GPIbα- and complement C3-dependent process, which was important for anti-bacterial CD8^+^ T cell responses (27). Since CD169^+^ macrophages are also an essential entry point for *Listeria monocytogenes* (28), platelets could be involved in the transfer of bacteria from the macrophage to the cDC1. We have previously shown that splenic CD169^+^ macrophages efficiently take up intravenously (i.v.) administered ganglioside GM3-containing liposomes and CD169-targeting antibody-antigen conjugates and subsequently collaborate with cDC1 to induce expansion of CD8^+^ T cells (29–32). Because of the similarities in the interaction between CD169^+^ macrophages and cDC1 during *Listeria monocytogenes* infection and after vaccination with GM3 liposomes, we hypothesized a possible role of platelets in GM3 liposome shuttle following liposomal vaccination.

The aim of the study was to investigate the interaction of platelets with liposomes, CD169^+^ macrophages and cDC1 and its implications for adaptive immune responses towards liposome-encapsulated ovalbumin (OVA) protein. We show that platelets bind liposomes, DNGR-1/Clec9a and CD169/Siglec-1 receptors *in vitro*. Furthermore, platelets associated with CD169^+^ macrophages and cDC1 *in vitro* and *in vivo*, where the presence of protease activated receptor 4 activating peptide (PAR4AP) and adjuvant (agonistic anti-CD40 antibody and poly (I:C)), respectively, boosted these interactions. Interestingly, the absence of platelets resulted in diminished liposome binding to cDC1 *in vivo*. Most importantly, platelet depletion, but not complement C3 deficiency, resulted in significantly diminished antigen-specific T cell responses leaving B cell responses unaffected. Mechanistically, while platelets did not affect the maturation status of the DCs, they influenced CCR7 expression levels by DCs and significantly contributed to CCL5 plasma levels. Collectively, our study provides robust evidence for platelet-CD169^+^ macrophage and platelet-DC cross-talk and the importance of platelets in induction of T cell responses in the context of i.v. liposomal vaccination. Our data guides future efforts in unveiling underlying mechanistic cues governing this process.

## 2 Materials and methods

### 2.1 Animals

C57Bl6/J WT mice were bred in-house and maintained in the animal facility of Amsterdam UMC (location VUmc) or purchased from Charles River. C3KO mice were bred in-house and maintained in the animal facility of Leiden UMC. Males and females between the age of 8 and 16 weeks were used for all experiments. All animals’ procedures were performed in accordance with Dutch government guidelines approved by Animal Experiment Committee (DEC) and Central Committee on Animal Experiments (CCD, ADV1140020171024).

### 2.2 Platelet isolation and PRP preparation

Peripheral blood was collected via cardiac puncture with a 27 G needle rinsed in sodium citrate into 2 ml eppendorfs containing sodium citrate (1:5), slowly rotated few times and stored at RT. Platelet rich plasma (PRP) was obtained by centrifugation at 180 g for 15 min (acceleration 7, break 2). Next, Acid Citrate Dextrose (ACD, in-house made) was added 1:5, PRP was slowly rotated few times and used for further *in vitro* analysis.

### 2.3 Liposome preparation

Liposomes were prepared using dry film extrusion method at the Department of Pharmaceutics, Faculty of Science, Utrecht University as described previously (29). In brief, egg phosphatidylcholine (EPC)-35 (Lipoid), egg phosphatidylglycerol (EPG)-Na (Lipoid) and Cholesterol (Sigma-Aldrich) (3.8:1:2.5) were mixed in chloroform/ethanol with 0.1 mol% of the lipophilic fluorescent tracer DiD (1′ -dioctadecyl-3,3,3′,3′ -tetramethyl indodicarbocyanine, Life Technologies) and where indicated with 3 mol% GM3 ganglioside (monosialodihexosylganglioside) (Avanti Polar Lipids). After evaporation of the organic solvent in a rotary evaporator, the obtained lipid film was hydrated in 1 mg/ml ovalbumin solution (OVA, Calbiochem). Next, the solution was extruded five times through stacked 400 nm and 200nm filters using high-pressure extruder and ultracentrifuged two times at 55000 rpm for 1 h at 4°C to pellet the liposomes. Next, the supernatant containing non-encapsulated OVA (and GM3 ganglioside) was removed and the pelleted liposomes were resuspended in HEPES buffer pH 7.5 containing antibiotics (50 U/ml penicillin and 50 µg/ml streptomycin, Lonza) by vortexing. Physicochemical characterization of obtained liposomal preparations included measurement of polydispersity index, mean size and zeta potential and was performed using the Zetasizer Nano ZSP instrument (Malvern Instruments, Malvern, UK).

### 2.4 Liposome, CD169/Siglec-1 Fc and DNGR-1/Clec9a binding

For liposome binding, PRP was first activated with 0.2 mg/ml (Ala1)-PAR-4 (1–6) amide (mouse) trifluoroacetate salt (protease activated receptor 4 activating peptide, PAR4AP, Bachem) for 30 min at 37°C and then stained with surface antibody mix containing 100 µM of liposomes for 30 min at RT. Next, stained PRP was washed with PBS, centrifuged at 800 g for 2 min (acceleration 7, break 2) and fixed with 1% PFA for 20 min at RT. Then PRP was washed, diluted in clean PBS and measured on Attune flow cytometer (Thermo Fisher) with the following acquisition settings: speed 100 µl/min, acquisition volume 115 µl, stopping gate 20,000 CD41^+^ events. Two-step clean cycle (Contrad 50% and water) was performed between the samples to prevent sample to sample carryover.

For Siglec-1 Fc binding, first 1.1 µg mouse Siglec-1 Fc protein or mutant (R97A mutation) Siglec-1 Fc incapable of sialic acid binding (33) was pre-complexed with 1.5 µg goat anti-human IgG (H+L) AF488 (Thermo Fisher) for 1 h on ice. In the meantime, PRP was activated with 0.2 mg/ml PAR4AP (Bachem) for 30 min at 37°C and subsequently incubated with 10 µg/ml of FcR block (2.4G2 clone, in-house made) for 15 min at RT. Next, PRP was stained with surface antibody mix containing pre-complexed Siglec-1 Fc-AF488 for 1h at RT, washed with PBS, centrifuged at 800 g for 2 min (acceleration 7, break 2) and fixed with 1% PFA for 20 min at RT. Then washed PRP was diluted in clean PBS and measured on Fortessa flow cytometer (BD Biosciences). The threshold, FSC and SSC were adjusted for small particle measurement.

For DNGR-1/Clec9a Fc binding, PRP was first activated with 0.2 mg/ml PAR4AP (Bachem) for 30 min at 37°C and subsequently incubated with 10 µg/ml of FcR block (2.4G2 clone, in-house made) for 15 min at RT. Next, PRP was stained with 2 µg/ml recombinant mouse Clec9A-Fc (R&D Systems) for 30 min at RT, washed with PBS, centrifuged at 800 g for 2 min (acceleration 7, break 2), stained with 2.5 µg/ml goat anti-mouse IgG2a biotin (Invitrogen), washed and incubated with 3 µg/ml Streptavidin-AF488 (Thermo Fisher) for 30 min at RT. After washing, stained PRP was fixed with 1% PFA for 20 min at RT. Then PRP was washed, diluted in clean PBS and measured on Fortessa flow cytometer (BD Biosciences). The threshold, FSC and SSC were adjusted for small particle measurement.

### 2.5 Complement C3 deposition

MaxiSorp ELISA plates (NUNC, Denmark) were coated overnight with 50 μmol liposomes in coating buffer (pH 9.2) or 40 μg/ml of LPS (Sigma-Aldrich) in PBS at 4°C. The following morning the plates were washed with PBS/0.02% Tween-20 and incubated for 1 h at RT on a shaking plate with mouse serum 1:40 or 1:30 diluted in Veronal Buffer (1.8 mM sodium barbital and 3.1 mM barbituric acid, pH 7.3–7.4; VB) supplemented with 10 mM MgCl2, 3 mM CaCl2, 0.05% Gelatin and 0.1% Tween-20. Next washed with PBS/0.02% Tween-20 plates were incubated for 1 h at RT on a shaking plate with purified C3b/c-bt antibody in High-performance ELISA buffer (HPE) (Sanquin Reagents). Following washing, poly-HRP (Sanquin Reagents) was added for 30 min at RT on a shaking plate. The ELISA was developed with 100 μg/ml of TMB (Sigma-Aldrich) in 0.11 M sodium acetate containing 0.003% H2O2 (Merck) and the reaction was stopped with 100 µL H2SO4. The absorbance was measured at 450 nm and corrected for the background absorbance at 540 nm with BioTek microtiter plate reader.

### 2.6 Platelet depletion

Platelets were depleted using a two-step protocol where mice were first injected intraperitoneally (i.p.) with 50 µl of anti-mouse thrombocyte antiserum (polyclonal) (rabbit serum) (Cedarlane) and the following day mice were injected intravenously (i.v.) in the tail vein with 1,75 ug/g mouse of anti-mouse GPIbα (monoclonal) (Emfret Analytics). To confirm platelet removal, peripheral blood was collected into Eppendorf tubes containing sodium citrate (1:5), diluted 10x in 0.5% BSA/PBS (from here on referred to as platelet buffer), stained with anti-CD41 for 30 min at RT and measured on Fortessa flow cytometer (BD Biosciences).

### 2.7 Liposome immunization and spleen digestion

Mice were injected i.v. in the tail vein with 93 nM of liposomal solution and 25 µg of anti-CD40 (1C10 clone in-house made) and 25 µg of poly(I:C) (*In vivo*gen) in PBS.

For myeloid cell analysis, spleens were collected at 16 h post immunization (p.i.) and digested as described previously (29). In brief, mechanistically dissociated spleen tissue was incubated with 4 mg/ml Lidocaine (Sigma-Aldrich), 2 WU/ml Liberase TL (Roche) and 50 µg DNase I (Roche, Germany) under continuous stirring for 15 min at 37°C. After enzymatic digestion, cold RPMI-1640 (Gibco, Life Technologies) supplemented with 10% heat-inactivated FCS (Biowest), 10 mmol EDTA, 20 mmol HEPES and 50 μM 2-mercaptoethanol was added and the cell suspension was further incubated for 10 min at 4°C under continuous stirring. Finally, pelleted splenocytes were exposed to ammonium-chloride-potassium (ACK) lysis buffer (in-house made) and filtered through 100 µm filter to obtain single cell suspension. For T/B cell analysis, spleens were collected at day 7 p.i. and digested as described previously (29). In brief, mechanistically dissociated spleen tissue was filtered through 100 µm filter and incubated with ACK lysis buffer.

### 2.8 Flow cytometry

For direct identification of splenic immune cell populations and germinal center B cell analysis, surface staining protocol was performed. In brief, single cell suspensions were first incubated with 10 µg/ml of FcR block (2.4G2 clone, in-house made) and Fixable Viability Dye (eBioscience) for 15 min on ice and subsequently stained with appropriate surface antibody mix (**Table 1**) for 30 min on ice. Following washing, stained cells were fixed using 2% paraformaldehyde (PFA) for 20 min at 4°C and measured on Fortessa (BD Biosciences). Obtained flow cytometry data was analyzed using FlowJo V10 software.

**Table 1:** List of antibodies/fluorescent reagents used for flow cytometry.

Antigen/reagent	Fluorochrome	Clone	Company	Panel
**CD169**	Alexa Fluor 488	SER-4	in-house made	Myeloid/ Splenocyte:platelet co-culture/ DC maturation
**Sirp1α**	Alexa Fluor 700	P84	Biolegend	
**F4/80**	PE-CF594	T45-2342	BD Biosciences	
**XCR1**	BV421	ZET	BD Biosciences	
**CD11c**	BV650	HL3	BD Biosciences	
**I-A/I-E**	BV510	M5/114.15.2	eBioscience	
**CD62P**	BV711/ PE-Cy7	RB40.34/ RMP-1	BD Biosciences/ Biolegend	
**CD41**	PE/BV605	MWReg30	Biolegend	
**CD40**	biotin	1C10	in-house made	DC maturation
**CD80**	PE	16-10A1	Immunotools	
**CD86**	PE-Cy7	53-6.7	BD Biosciences	
**CCR7**	PE-Cy5	4B12	Biolegend	
**PD-L1**	BV785	B7-H1	Biolegend	
**CD8a**	APC	53-6.7	BD Biosciences	CD8^+^ T cell tetramer staining
**CD44**	FITC	KM81	Immunotools	
**H-2K^b^/SIINFEKL**	PE tetramer	N/A	LUMC, Leiden	
**B220**	BV510	RA3-6B2	Biolegend	Germinal B cell staining
**CD38**	PE	90/CD38	BD Biosciences	
**GL7**	PE-Cy7	GL-7	Biolegend	
**OVA**	Alexa Fluor 488	N/A	Invitrogen	
**CD11a**	FITC	M17/4	eBioscience	Re-stim intracellular IFNγ staining
**CD8a**	PE-Cy7	53-6.7	BD Biosciences	
**CD4**	PE	GK1.5	eBioscience	
**IFNγ**	APC	XMG1.2	eBioscience	

For analysis of antigen-specific T cell responses, following incubation with FcR block and Fixable Viability Dye, splenocytes were stained with H-2Kb/SIINFEKL tetramers for 1 h at 37°C, washed and finally incubated with surface antibody mix. For re-stimulation with OVA peptides, splenocytes were incubated with MHC I-restricted OVA_257–264_ peptide (0.1 µg/ml) for 5 h or with the MHC II-restricted OVA_262–276_ peptide (100 µg/ml) for 24 h in the presence of GolgiPlug during the last 5 h (BD Biosciences). Following re-stimulation, surface stained and fixed cells were permeabilized with 0.5% Saponin (Sigma-Aldrich), washed and stained with anti-IFNγ for 30 min on ice. For analysis of antigen-specific B cell responses, following incubation with FcR block and Fixable Viability Dye, splenocytes were stained with surface mix containing fluorescently labelled OVA for 30 min on ice.

### 2.9 PRP and splenocyte co-culture

Prepared as described above PRP was first activated with 0.2 mg/ml PAR4AP) (Bachem) for 30 min at 37°C, washed and diluted in 1% BSA/PBS. Digested as described above (for myeloid cell analysis) splenocytes were co-incubated with activated PRP for 1 h at RT. Next the cells were washed, centrifuged at 1500 rpm for 5 min, surface stained, fixed and measured on Fortessa (BD Biosciences).

### 2.10 Cytokine analysis in serum

Blood was collected via heart puncture from liposome-immunized mice at 16 h p.i. and centrifuged at 1500 g for 10 min at 4°C to obtain serum that was stored at -20°C until the assay was performed. LEGENDplex immunoassay (Biolegend) was performed on thawed serum according to the manufacturer’s instructions. In brief, diluted serum and standard were incubated with beads and assay buffer for 2 h at RT. Following washing, the samples were incubated with the detection antibodies for 1h at RT and subsequently with Streptavidin-PE for 30 min at RT. Washed samples were measured on Fortessa (BD Biosciences). The data was analyzed using an online software tool provided by the manufacturer.

### 2.11 Anti-OVA Ig analysis in serum

Blood was collected via heart puncture from liposome-immunized mice at day 7 p.i. and centrifuged at 1500 g for 10 min at 4°C to obtain serum that was stored at -20°C until the assay was performed. MaxiSorp ELISA plates (NUNC) were coated with 5 µg/ml OVA (Sigma-Aldrich) in sodium phosphate buffer pH 6.5 (in-house made) and left o/n at 4°C. Next, the samples were washed with 0.05% Tween20/PBS, blocked with 1% BSA/PBS for 1 h at RT and incubated with serial dilutions of serum in 1% BSA/PBS for 2h at RT. After washing, rabbit anti-mouse Ig-HRP (Dako) was added for 1 h at RT, washed away and then 100 µg/ml of TMB (Sigma-Aldrich) substrate and 0.006% hydrogen peroxide in substrate buffer was added to develop the reaction. The absorbance was measured at 450 nm using a microplate absorbance spectrophotometer (Biorad). A cut-off value was set at 0,25. Measured OD values higher than or equal to the cut-off value were used to determine the corresponding antibody titers.

### 2.12 Statistical analysis

One-way ANOVA test with Tukey’s multiple comparison test was performed to determine statistical significance using GraphPad Prism software. Differences were considered significant when *p < 0.05, **p < 0.01. All values are expressed as ± SEM with individual mice showed.

## 3 Results

### 3.1 Platelets bind liposomes *in vitro* in an activation-independent manner

**Figure 1:**
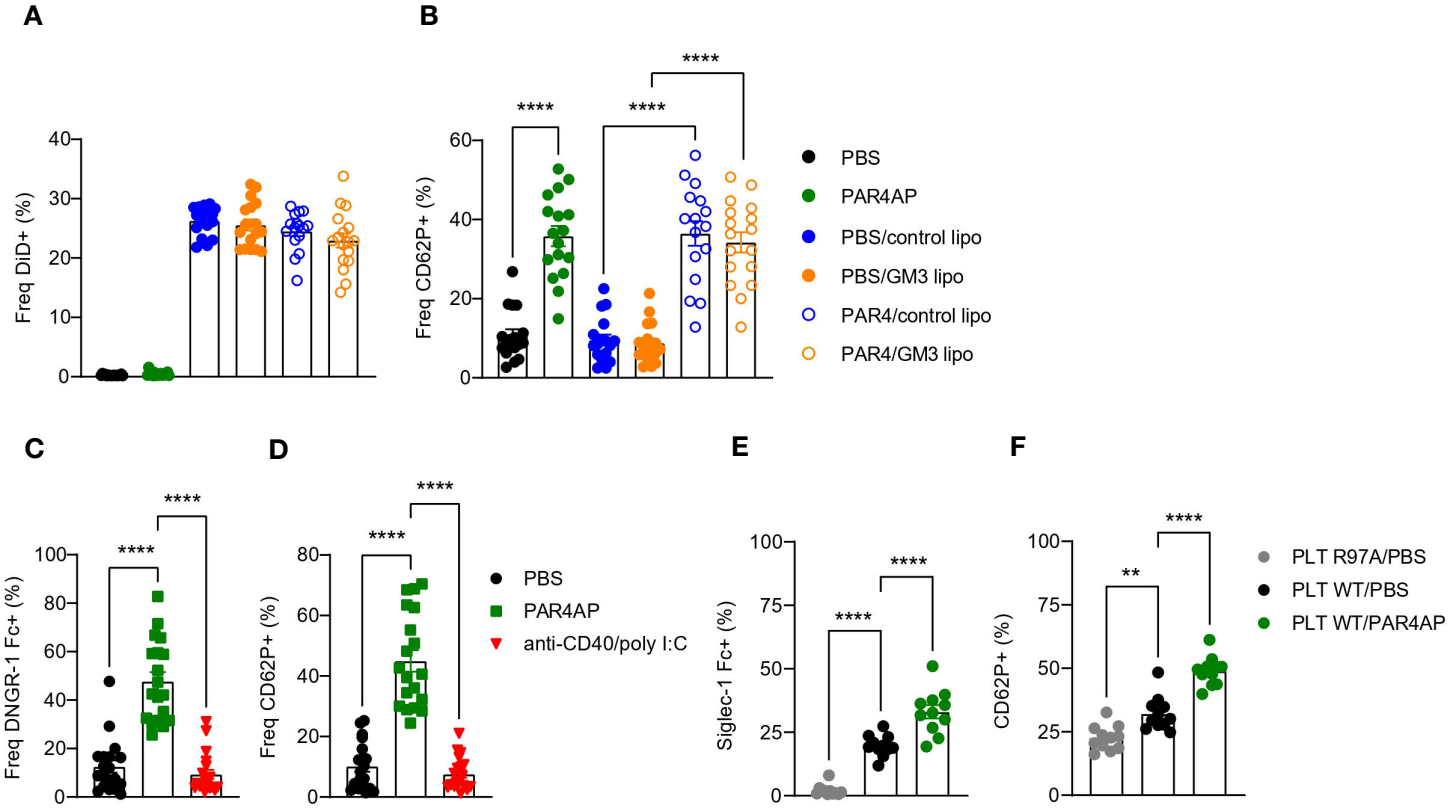
Platelets bind to control and GM3 ganglioside liposomes, DNGR-1 Fc and Siglec-1/CD169 Fc *in vitro*. **(A, B)** Platelets were pre-incubated with PBS or PAR4AP (0.2 mg/ml) and incubated with DiD labelled control or GM3 liposomes (100 µM) for 30 min at RT. **(A)** Percentage of DiD^+^ events within CD41^+^ platelets determined by flow cytometry. **(B)** Activation of liposome-bound platelets from **(A)** The data are from three independent experiments (n = 18). **(C, D)** Platelets were pre-incubated with PBS, PAR4AP (0.2 mg/ml) or anti-CD40/poly(I:C) adjuvant (5 µg/ml) for 30 min at RT and incubated with DNGR-1 Fc (2 µg/ml) for 30 min at RT. **(C)** Percentage of DNGR-1 Fc^+^ events within CD41^+^ platelets determined by flow cytometry. **(D)** Activation of platelets from **(C)** The data are from three independent experiments (n = 13). **(E, F)** Platelets were pre-incubated with PBS or PAR4AP (0.2 mg/ml) for 30 min at RT and incubated with pre-complexed Siglec-1/CD169 Fc-AF488 (1.1 µg/ml) for 1h at RT. **(E)** Percentage of Siglec-1 Fc^+^ events within CD41^+^ platelets determined by flow cytometry. **(F)** Activation of platelets from **(E)** The data are from one experiment (n = 12). R97A, Siglec-1/CD169 mutant; PLT, platelet. Each symbol represents PRP collected from one mouse. Error bars indicate mean ± SEM. Statistical analysis one way ANOVA with Tukey’s multiple comparison test (**p < 0.01, ****p < 0.0001).

Platelets are equipped with various receptors that enable binding and engulfment of small particles such as bacteria and viruses, but also liposomes. Such interactions mostly result in platelet activation, degranulation and clearance by the liver (34). To test our hypothesis that platelets bind control and/or GM3 liposomes and to evaluate the subsequent effect of liposomes on platelet activation, we incubated control and GM3 liposomes with resting and PAR4AP-activated platelets and quantified liposome-platelet contacts (**Figures 1A, B**). We found that approximately 25% of platelets bound control and GM3-containing DiD-labelled liposomes, as quantified by platelet-associated DiD signal, suggesting that platelets do not exhibit a preference for either of the nanoparticles (**Figure 1A**). Stimulation with PAR4AP, a potent platelet activator, did not further enhance platelet-liposome interaction, indicating that it occurs in an activation-independent manner. Upon activation, platelets translocate P-selectin (CD62P) to the their membrane (**Figure 1B**), but exposure of platelets to control or GM3 liposomes did not alter CD62P expression. These findings demonstrate that control and GM3 liposomes bind to platelets to a similar extent in an activation-independent manner *in vitro* and that such interaction does not influence the activation state of the platelets.

### 3.2 PAR4AP-activated platelets bind to DNGR-1/Clec9a Fc *in vitro*

Platelets have been previously shown to act as a shuttle for bacteria and carry them to DCs (27). Therefore, after confirming that liposomes and platelets interact *in vitro*, we wondered whether platelets would use a similar mechanism following i.v. liposome immunization and deliver the liposomes to the DCs. Activated platelets, expose F-actin which is known to act as substrate for DNGR-1 (35, 36). DNGR-1/Clec9A is a receptor specifically expressed by cDC1 that facilitates cross-presentation of dead-cell associated antigens. Therefore, we hypothesized that upon i.v. co-injection of GM3 liposomes and anti-CD40/poly(I:C) adjuvant, liposome-associated platelets become activated by the adjuvant and bind to cDC1 via DNGR-1. To test this, resting or adjuvant-stimulated platelets were incubated with DNGR-1 Fc conjugate to determine binding (**Figures 1C, D**). PAR4AP was used a positive control for platelet activation. While PBS-treated platelets already exhibited some level of binding to DNGR-1, probably due to background activation, this was significantly increased and reached 50% upon platelet exposure to PAR4AP (**Figure 1C**). In contrast, incubation with the adjuvant used in our immunization studies, anti-CD40/poly(I:C), did not result in CD62P upregulation on platelets nor in DNGR-1 binding (**Figure 1D**). These findings clearly show that activated platelets bind to DNGR-1/Clec9a, but also reveal lack of platelet-activating capacity of anti-CD40/pol I:C *in vitro*.

### 3.3 Activated platelets exhibit enhanced binding to Siglec-1/CD169 Fc *in vitro*

We have previously demonstrated that splenic CD169^+^ macrophages efficiently take up liposomes containing sialylated ligands, such as GM3, as well as control liposomes from the blood circulation (29, 30, 37) and that these macrophages engage in a collaboration with the cDC1 subset in the spleen (29–32). Glycoproteins present on the surface of platelets are highly sialylated and could mediate the interaction between platelet-bound liposomes and CD169/Siglec-1. To examine this, we investigated the binding of Siglec-1/CD169 Fc to resting or PAR4AP-activated platelets (**Figures 1E, F**). Approximately 20% of platelets bound to Siglec-1 Fc, while no binding of platelets to mutant Siglec-1 Fc protein, with disrupted receptor-ligand binding due to a mutation, was observed. Unexpectedly, exposure to PAR4AP appeared to enhance the interaction between platelets and Siglec-1 Fc, illustrated by 30% of Siglec-1 Fc^+^ platelets. Previous reports have shown that upon activation, platelets translocate neuraminidase to their surface that subsequently cleaves off sialic acids, which could function as CD169 ligands, therefore we expected less Siglec-1 Fc binding after activation (38). Overall, this data indicates that platelets interact with Siglec-1 Fc *in vitro* and that this interaction is augmented upon PAR4AP stimulation.

### 3.4 PAR4AP and adjuvant enhance platelet-macrophage and platelet-DC interactions *in vitro* and *in vivo*

**Figure 2:**
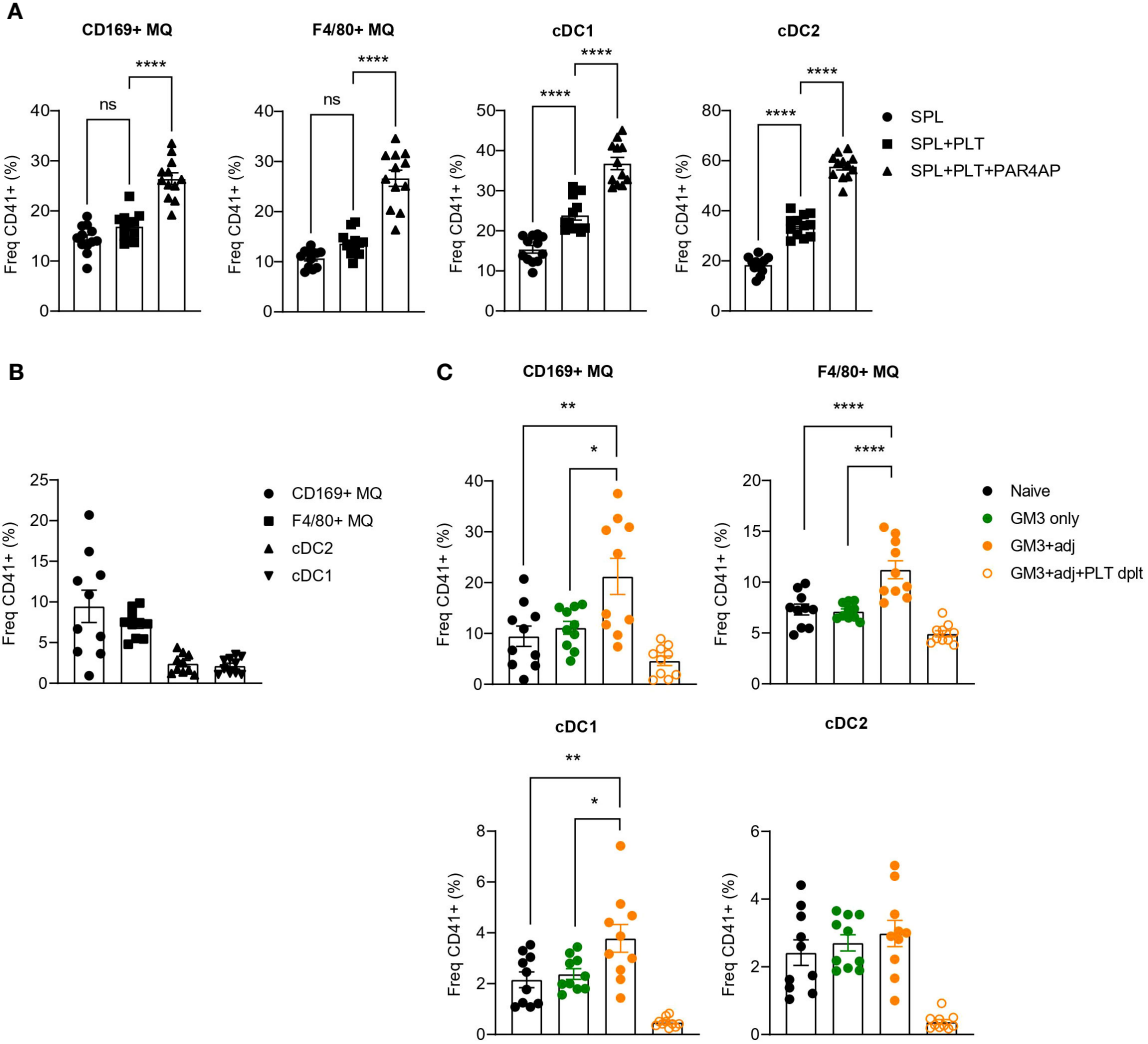
Increased binding of platelets to CD169^+^ macrophages and DCs revealed upon PAR4AP and adjuvant treatment. **(A)** Platelets were pre-incubated with PBS or PAR4AP (0.2 mg/ml) for 30 min at RT and co-cultured with splenic macrophages and DCs for 1 h at RT and the percentage of CD41^+^ platelet-bound cells were determined by flow cytometry. SPL, spleen; PLT, platelet. The data are from one experiment (n = 12). **(B, C)** Platelet proficient or depleted mice were injected i.v. with GM3 liposomes (93 nmoles) in the presence or absence of anti-CD40/poly(I:C) (each 25 µg/mouse) and the percentage of CD41^+^ platelet-bound cells in naïve mice **(B)** and immunized mice **(C)** was determined at 16 h p.i. by flow cytometry. GM3, GM3 liposomes; adj, adjuvant; PLT dplt, platelet-depleted. The data are from two independent experiments representative of three independent experiments (n = 15). Each symbol represents one mouse. Error bars indicate mean ± SEM. Statistical analysis one way ANOVA with Tukey’s multiple comparison test **(B)** (*p < 0.05, **p < 0.01, ****p < 0.0001). ns, not significant.

Our findings using recombinant receptors, demonstrated DNGR-1- and Siglec-1-binding capacity of platelets which indicated a possible interaction between platelets and CD169^+^ macrophages and cDC1s. To determine whether such interactions occur *in vitro*, we incubated resting or PAR4AP-activated platelets with splenocytes and quantified binding of platelets to DCs and macrophages after 1 h (**Figure 2A**). In line with the previous data, we observed interactions of platelets with CD169^+^ macrophages and cDC1, but also red pulp F4/80^+^ macrophages and cDC2. Moreover, presence of PAR4AP appeared to enhance the binding of platelets to mentioned myeloid cell populations.

To confirm these interactions *in vivo*, we immunized mice with OVA-containing GM3 liposomes co-injected or not with adjuvant, and evaluated the binding of platelets to DCs and macrophages 16h post injection (p.i.) (**Figures 2B, C**). Similarly to what we observed *in vitro*, platelets associated with CD169^+^ macrophages, F4/80^+^ macrophages and DCs already in naïve mice (**Figure 2B**). While these interactions were not affected by administration of GM3 liposomes, the presence of the adjuvant appeared to enhance platelet binding to CD169^+^ macrophages, red pulp macrophages and cDC1, but not to cDC2 (**Figure 2C**). Overall, these results demonstrate that platelets engage with macrophages and DCs *in vitro* and *in vivo* and that the presence of the PAR4AP or adjuvant, respectively, augments these interactions.

### 3.5 Enhanced liposome uptake by F4/80^+^ macrophages and cDC1 in the presence of platelets

**Figure 3:**
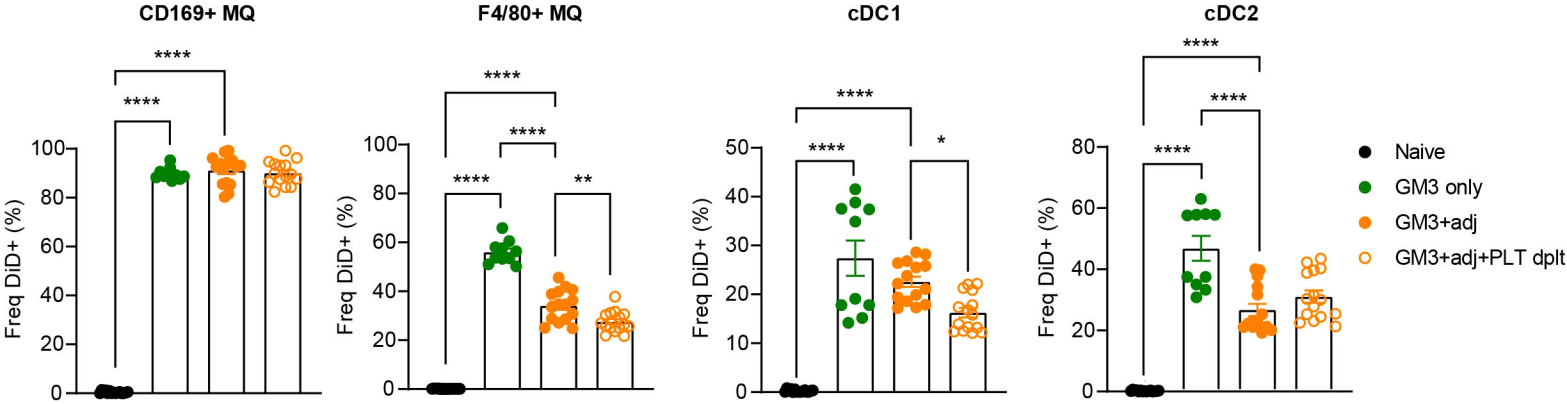
Presence of platelets enhances GM3 liposome uptake by F4/80^+^ macrophages and cDC1 *in vivo*. Platelet proficient and platelet-deficient mice were injected i.v. with GM3 liposomes (93 nmoles) in the presence or absence of anti-CD40/poly(I:C) (each 25 µg/mouse) adjuvant and the percentage of GM3 liposome-bound DiD^+^ cells was determined at 16 h p.i. by flow cytometry. GM3, GM3 liposomes; adj, adjuvant; PLT dplt, platelet-depleted. The data are from two independent experiments representative of three independent experiments (n = 10). Each symbol represents one mouse. Error bars indicate mean ± SEM. Statistical analysis one way ANOVA with Tukey’s multiple comparison test (*p < 0.05, **p < 0.01, ****p < 0.0001).

Enhanced binding of platelets to macrophages and cDC1s in liposome-immunized mice and costaining of CD41 and liposomes in macrophages and DCs (**Figure S1**) prompted us to address the role of platelets in the liposome uptake by these myeloid cell populations. To test this, we depleted the platelets using a combination of a polyclonal rabbit anti-mouse platelet serum and a monoclonal anti-GPIbα antibody prior to vaccination with OVA-containing GM3 liposomes and adjuvant, and analyzed DiD signal associated with splenic myeloid populations 16h p.i. (**Figures S2**, **3**). In agreement with our previous work (29), CD169^+^ macrophages were superior to red pulp F4/80^+^ macrophages and DCs in capture of liposomes containing the CD169 ligand GM3. In the absence of platelets, F4/80^+^ macrophages and cDC1 exhibited significantly lower liposome uptake, while liposome uptake by CD169^+^ macrophages and cDC2 was largely unaffected or slightly increased, respectively (**Figure 3A**). Interestingly, DiD geometric mean fluorescence intensity (gMFI) was decreased on cDC1 and increased on cDC2 in platelet-depleted animals, suggesting a platelet-induced shift in liposome capture from cDC1 to cDC2 (**Figure S3**). Together, these data indicate that platelets contribute to liposome capture by red pulp macrophages and cDC1.

### 3.6 Diminished T cell but not B cell responses in the absence of platelets

**Figure 4:**
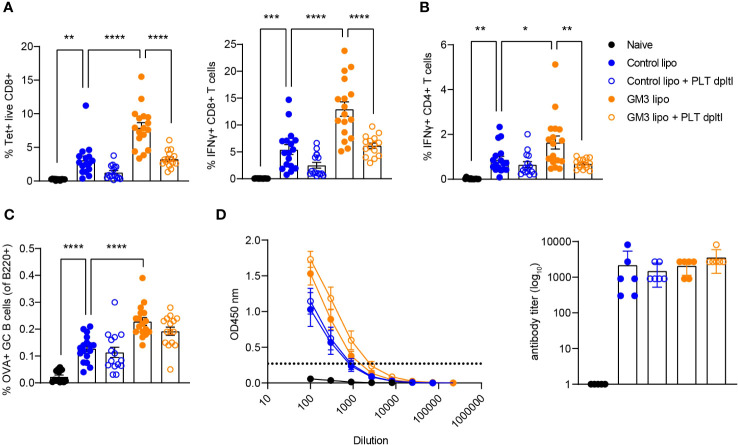
Presence of platelets supports T cell responses following liposomal vaccination. Platelet proficient and platelet-deficient mice were injected i.v. with GM3 liposomes (93 nmoles) in the presence of anti-CD40/poly(I:C) (each 25 µg/mouse) adjuvant and the immune responses were evaluated on day 7 p.i. by flow cytometry. **(A)** Percentage of H-2Kb-SIINFEKL-tetramer^+^ CD8^+^ T cells determined directly and the percentage of IFNγ producing CD8^+^ T cells determined after *in vitro* peptide re-stimulation. **(B)** Percentage of IFNγ producing CD4^+^ T cells determined after *in vitro* peptide re-stimulation. **(C)** Percentage of OVA-specific germinal center B cells determined gated as B220^+^CD38^-^GL7^+^OVA^+^. **(D)** Detection of OVA-specific Ig determined by ELISA. The dotted line indicates the cut-off value for antibody titer determination. The data are from three independent experiments (n = 15-17). Each symbol represents one mouse. Error bars indicate mean ± SEM **(A–C)** or geometric mean ^+^ 95% CI **(D)**. Statistical analysis one way ANOVA with Tukey’s multiple comparison test (*p < 0.05, **p < 0.01, ***p < 0.001, ****p < 0.0001).

We previously showed that cDC1 are essential for CD8^+^ T cell priming after liposomal vaccination (29, 30). Since we observed platelet binding to DNGR-1/Clec9a Fc *in vitro* and to cDC1 *in vivo*, and diminished liposome capture by cDC1 in the absence of platelets, we assessed the role of platelets in induction of adaptive immunity in context of liposomal vaccination. To this end, we first depleted the platelets as described above, and two days later we i.v. injected OVA-encapsulated control (lacking GM3) and GM3 liposomes and adjuvant. On day 7 following i.v. immunization, OVA-specific T and B cell responses in the spleen were evaluated (**Figure 4**). We detected significantly lower frequencies of tetramer^+^ CD8^+^ T cells and IFNγ-producing CD8^+^ T cells upon re-stimulation in both control liposome- and GM3 liposome- vaccinated animals, in the absence of platelets (**Figure 4A**). Similar to our findings for CD8^+^ T cells, the magnitude of CD4^+^ T cell response was also negatively affected by platelet depletion, illustrated by decreased population of IFNγ^+^ CD4^+^ T cells found liposome- immunized mice (**Figure 4B**). The presence of platelets appeared irrelevant for induction of B cell responses, as we observed no change in the frequency of germinal center B cell population, or total OVA immunoglobulin levels in the mouse serum in platelet-depleted condition (**Figures 4C, D**). These results clearly demonstrate the importance of platelets in generation of robust cytotoxic and helper T cell responses, but not B cell responses, induced after liposome immunization.

### 3.7 Liposomes induce complement activation *in vitro*, but the absence of complement C3 does not affect T and B cell responses stimulated by liposome immunization *in vivo*

**Figure 5:**
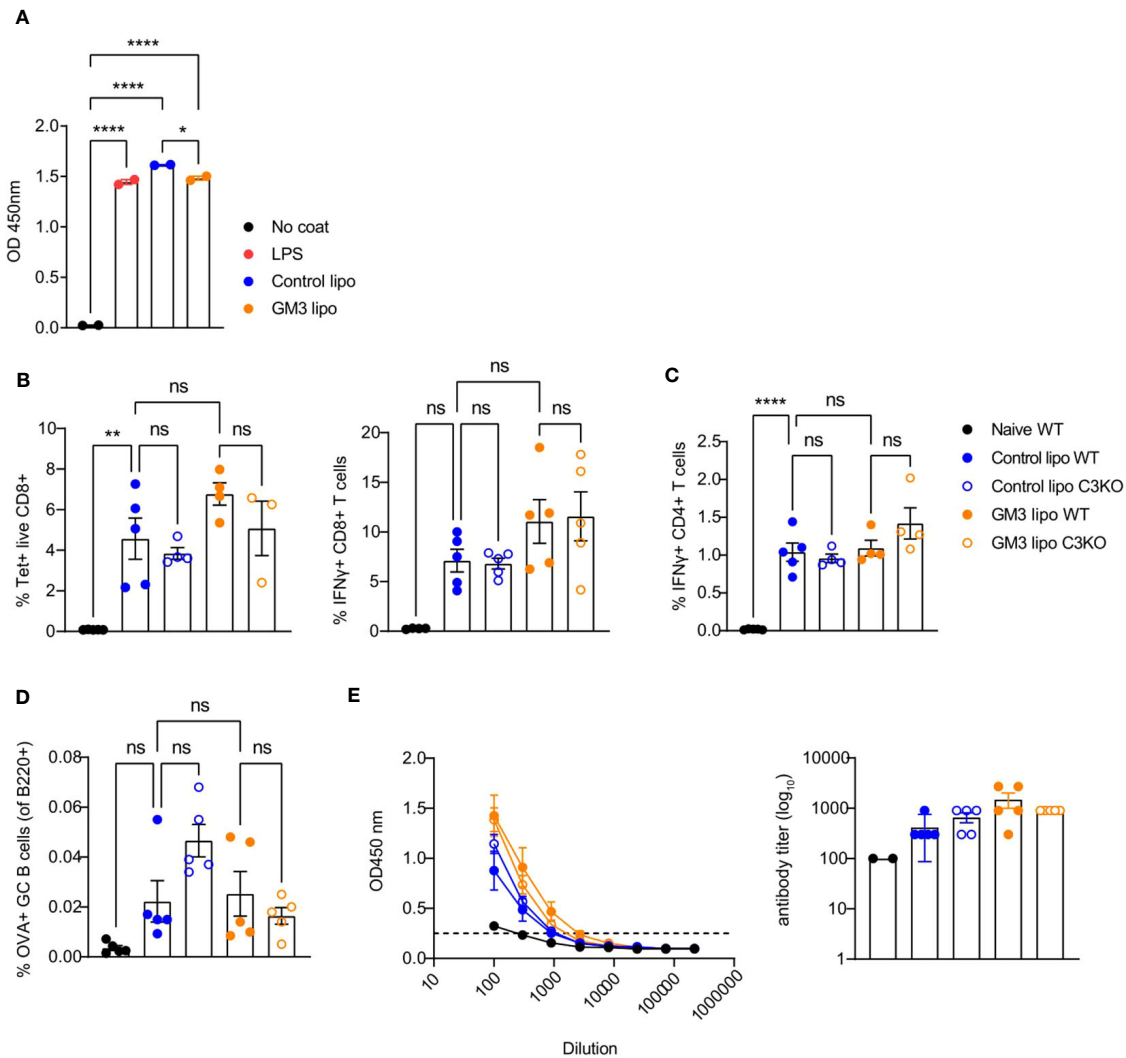
Control and GM3 liposomes activate complement C3 *in vitro* but complement C3 is not important for liposome-induced immune responses *in vivo*. **(A)** Control and GM3 liposomes were coated on an ELISA plate and incubated with mouse serum. Complement C3 deposition that was visualized by OD absorbance. The data is from one experiment representative of three. Each symbol represents one sample. **(B–E)** WT and complement C3-deficient mice were injected i.v. with control and GM3 liposomes (93 nmoles) in the presence of anti-CD40/poly(I:C) (each 25 µg/mouse) adjuvant and the immune responses were evaluated on day 7 p.i. by flow cytometry. **(B)** Percentage of H-2Kb-SIINFEKL-tetramer^+^ CD8^+^ T cells determined directly and the percentage of IFNγ producing CD8^+^ T cells determined after *in vitro* peptide re-stimulation. **(C)** Percentage of IFNγ producing CD4^+^ T cells determined after *in vitro* peptide re-stimulation. **(D)** Percentage of OVA-specific germinal center B cells determined gated as B220^+^CD38^-^GL7^+^OVA^+^. **(E)** Detection of OVA-specific Ig determined by ELISA. The dotted line indicates the cut-off value for antibody titer determination. The data is from one experiment. Each symbol represents one mouse. Error bars indicate mean ± SEM. Statistical analysis one way ANOVA with Tukey’s multiple comparison test (*p < 0.05, **p < 0.01, ****p < 0.0001). ns, not significant.

Liposomes have been shown to associate with plasma proteins including the complement system in the circulation (39, 40). Moreover, since complement C3 was demonstrated to mediate shuttling of platelet-bacteria complexes to cDC1 (27), we hypothesized a similar process to take place regarding the uptake of liposomes by CD169^+^ macrophages and cDC1. Therefore, we assessed the capacity of control and GM3 liposomes to activate the complement system by measuring complement element 3 (C3) deposition in an ELISA-based assay using mouse serum (**Figure 5A**). Complement C3 is a key protein required for all complement activation pathways to occur (41).

Both control as well as GM3 liposomes resulted in mouse complement C3 deposition that was of similar magnitude to the complement activation induced by LPS. Encouraged by these results we investigated the importance of complement C3 for induction of adaptive immunity in our vaccination platform. To this end, we immunized WT and C3KO mice with control and GM3 liposomes co-injected with adjuvant and evaluated T and B cell responses 7 days after vaccination (**Figures 5B–E**).

We observed no significant differences in frequencies of antigen-specific CD8^+^ and CD4^+^ T cells between WT and C3KO animals immunized with control and GM3 liposomes. In addition, liposome-vaccinated C3KO mice did not exhibit a decreased capacity in generating OVA^+^ germinal center B cells when compared to the WT counterpart. Interestingly, in the control liposome group, the magnitude of B cell responses appeared higher in C3KO animals than in WT animals. Together, these results demonstrate that both control and GM3 liposomes activate complement *in vitro*, but complement C3 is not essential for the induction of adaptive immunity upon i.v. administration of control and GM3 liposomes.

### 3.8 Platelets do not influence expression of co-stimulatory markers, but affect CCR7 expression by DCs

**Figure 6:**
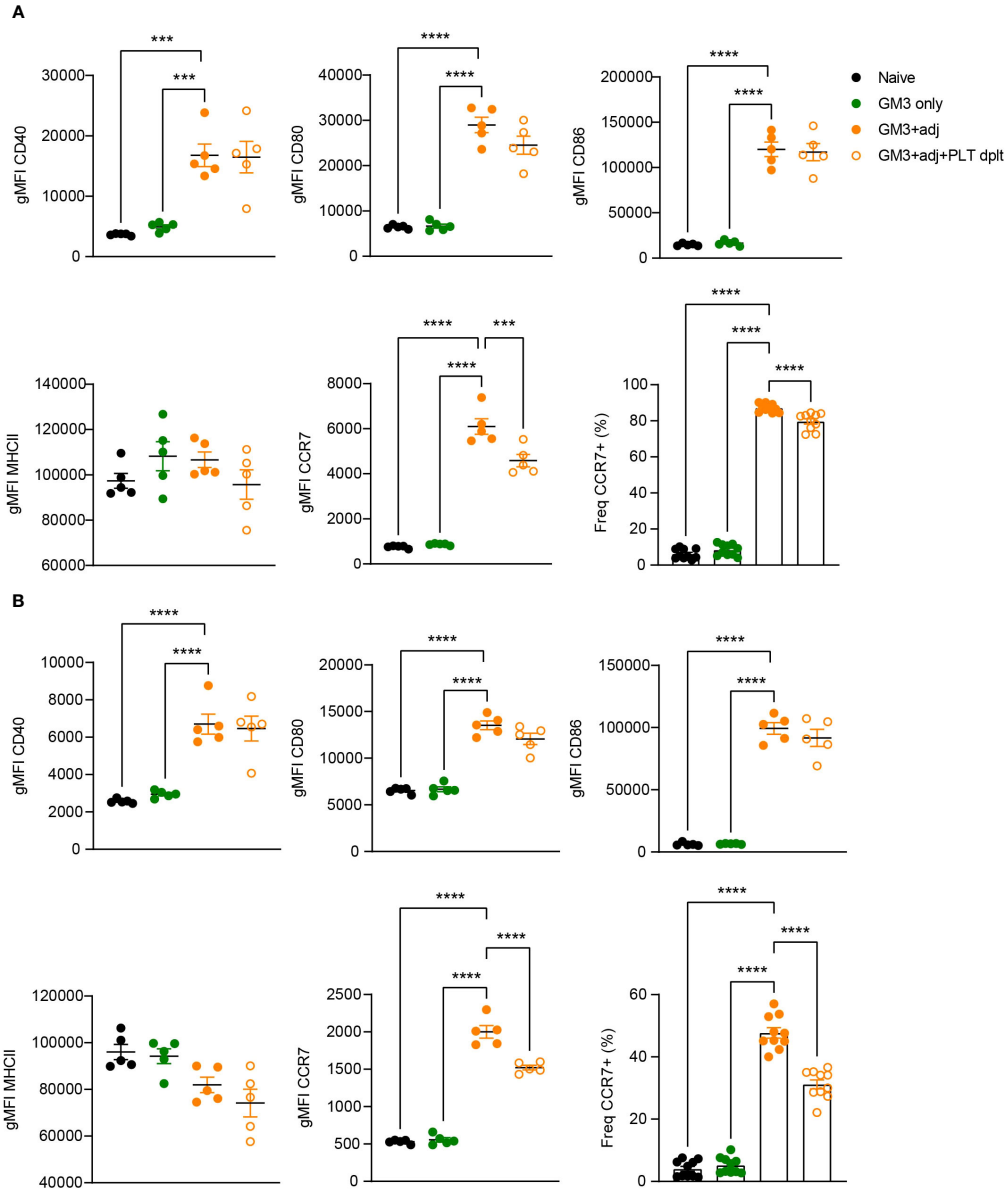
Expression of co-stimulatory markers by DCs is not affected by platelets. Platelet proficient and platelet-deficient mice mice were injected i.v. with GM3 liposomes (93 nmoles) in the presence or absence of anti-CD40/poly(I:C) (each 25 µg/mouse) adjuvant and gMFI of CD40, CD80, CD86, MHCII and CCR7 (also percentage) was determined at 16 h p.i. by flow cytometry on cDC1 **(A)** and cDC2 **(B)**. GM3, GM3 liposomes; adj, adjuvant; PLT dplt, platelet-depleted. The data shown as gMFI are from one experiment representative of two independent experiments (n = 5). The data shown as frequency are from two independent experiments (n = 10). Each symbol represents one mouse. Error bars indicate mean ± SEM. Statistical analysis one way ANOVA with Tukey’s multiple comparison test (***p < 0.001, ****p < 0.0001).

Platelet-derived CD40L has been previously demonstrated to induce DC maturation (17, 21, 25). Therefore, we next investigated the maturation status of DCs isolated from platelet-proficient or -deficient mice that were immunized with GM3 liposomes and adjuvant (**Figure 6**). Analysis of MHC class II, CD40, CD80 and CD86 expressed by cDC1 and cDC2 at 16h p.i., revealed no changes between platelet-depleted and non-depleted groups while exposing a strong adjuvant-dependence. However, we did observe a significant decrease in frequency of CCR7-expressing cDC1 and cDC2 as well as diminished CCR7 expression levels (gMFI), in the absence of platelets, which may affect their capacity to home to the T cell zone and to prime T cells. Taken together these results indicate that following co-injection of GM3 liposomes and adjuvant, platelets do not influence the co-stimulatory capacity of DCs, but they do affect their CCR7 expression.

### 3.9 Diminished serum CCL5 levels in the absence of platelets

**Figure 7:**
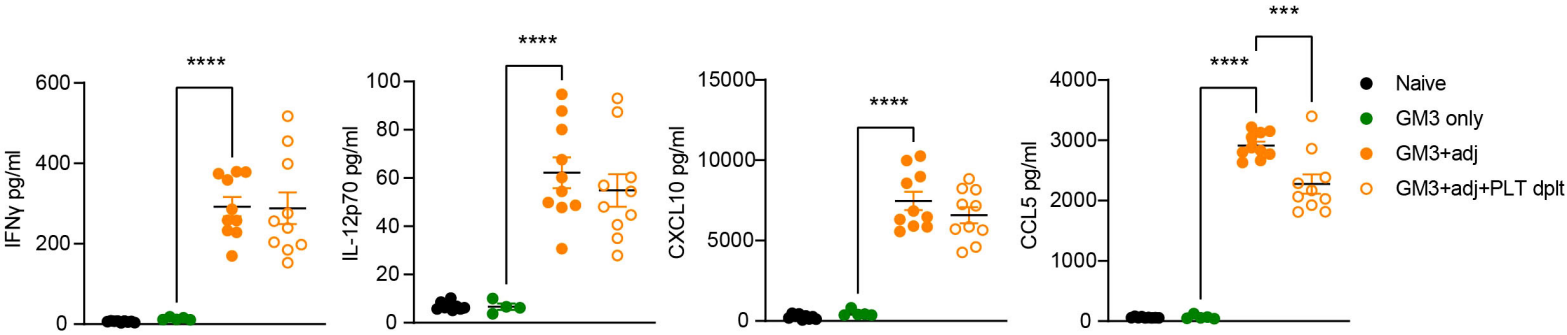
Platelet-depleted animals exhibit decreased CCL5 levels while IFNγ, IL-12p70 and CXCL10 remain unaffected. Platelet proficient and platelet-deficient mice were injected i.v. with GM3 liposomes (93 nmoles) in the presence or absence of anti-CD40/poly(I:C) (each 25 µg/mouse) adjuvant and IFNγ, IL-12p70, CXCL10 and CCL5 secretion in serum was measured at 16 h p.i. using LEGENDplex by flow cytometry. GM3, GM3 liposomes; adj, adjuvant; PLT dplt, platelet-depleted. The data are from two independent experiments (n = 10). Each symbol represents one mouse. Error bars indicate mean ± SEM. Statistical analysis one way ANOVA with Tukey’s multiple comparison test (***p < 0.001, ****p < 0.0001).

To further characterize the contribution of platelets to the observed T cell activation, we determined cytokine levels in the serum of platelet-depleted or proficient mice, collected at 16h p.i. (**Figure 7**). Analysis of IFNγ, IL-12p70 and CXCL10 revealed no changes in both of these Th1-associated cytokines and chemokine after platelet depletion. We also quantified presence of CCL5, a chemokine abundantly present in α-granules, which is released upon platelet activation and its receptor is expressed by effector and memory T cells. We observed high levels of CCL5 in the serum after injection with adjuvant, which were significantly reduced in the absence of platelets. Taken together this data shows that platelets do not influence IFNγ, IL-12p70 and CXCL10 levels, but they do contribute to the increase in CCL5 serum levels in liposome-immunized mice, and thereby could affect T cell responses.

## 4 Discussion

Owing to the plethora of surface-expressed receptors and granule-contained soluble mediators, combined with their high abundance in blood, platelets have been described as immune sentinels alarming other immune cells about the ongoing insult (42). The purpose of this study was to address the role of platelets in liposome-induced adaptive immunity and to characterize platelet interactions with liposomes and multiple antigen presenting cell (APC) types such as CD169^+^ macrophages, red pulp macrophages and DCs.

We demonstrate that platelets bind liposomes *in vitro* and that such interaction does not lead to platelet activation nor is platelet activation-dependent. Early reports have described a transient interaction between platelets and liposomes resulting in thrombocytopenia, the extent of which was dependent on physicochemical properties of the liposomes (43–47). Some studies demonstrated increased platelet aggregation upon exposure to ligand-bearing liposomes such as PEG or Lewis^x^ (48). Indeed, the surface of the nanoparticles is an important determining factor for interactions with platelets (49, 50). Nevertheless, in the current study we show that platelets are equally capable of binding GM3 liposomes and control liposomes.

Constantinescu and colleagues (47) noted increased binding of platelets to liposomes in the presence of plasma proteins, advocating for the role of plasma proteins in mediating such contacts. Complement factor C3b was identified as responsible for platelet-liposome microaggregates in rats (51). In fact, complement opsonization of bacteria has been previously reported to mediate bacteria entrapment by platelets and delivery to cDC1 and subsequent T cell priming (27, 52, 53). It is possible that both liposomes and bacteria share complement-dependency for platelet interaction. Our *in vitro* data indicate that both control and GM3 liposomes activate complement system and cause complement C3 deposition, a central protein important for all three complement activation pathways. Although we did not evaluate the effect of C3 on liposome binding to platelets, our *in vivo* data excludes C3 as an essential contributor to liposome-induced immune responses.

A study by Li et al. (54) described large platelet aggregates in close proximity of CD169^+^ macrophages in the spleen, which were diminished upon macrophage depletion. Also, Nicolai and colleagues (55) recently showed uptake of platelet-adenovirus vaccine aggregates by splenic marginal zone macrophages. We have previously demonstrated that CD169^+^ macrophages transfer antigen to cDC1 and thereby stimulate CD8^+^ T cell responses after immunization with different vaccines, including liposomes (29–32). Interesting, a similar dependency on both CD169^+^ macrophages and cDC1 was described for *Listeria monocytogenes* infection (28, 56). In addition, Verschoor et al. reported that platelets were also essential for platelet-mediated *Listeria monocytogenes* delivery to cDC1 (27). Based on these studies, we postulated that platelets could shuttle liposomes from CD169^+^ macrophages to cDC1 in an analogous manner as *Listeria monocytogenes*. Furthermore, we hypothesized that binding of CD169 to sialylated platelet glycoproteins and/or binding of DNGR-1/Clec9a to platelet actin would mediate interactions between platelets and CD169^+^ macrophages or cDC1, respectively.

Our main finding is that platelets contribute significantly to the T cell responses following liposomal challenge. Using a two-step platelet depletion protocol we showed a decrease in magnitude of both antigen-specific CD8^+^ and CD4^+^ T cell responses in the absence of platelets, while B cell responses were not affected. We have previously shown that T cell responses are dependent on cDC1 (29, 30), and indeed here we observed platelet association with cDC1 which was increased upon adjuvant injection. More importantly, the DiD levels in cDC1 were partially dependent on the presence of platelets. These observations suggest that platelets enhance the uptake of liposomes by cDC1 which is in line with the *Listeria monocytogenes* study by Verschoor et al. (27).

Furthermore, platelets could potentially stimulate cross-presentation via the interaction of platelet actin with DNGR-1 on cDC1. Accordingly, we observed DNGR-1 binding to activated platelets *in vitro*. Experiments evaluating binding of DNGR-1 to activated platelets identified F-actin as the principal (and so far, only) ligand for the receptor (35, 57). DNGR-1 ligation was demonstrated to be redundant for antigen uptake, however non-redundant for antigen cross-presentation and CD8^+^ T cell induction in the context of multiple viral infections (31, 58–60). It was shown that DNGR-1 together with endocytosed cargo is directed into recycling compartments where the antigen is retrieved and thus promotes efficient cross-presentation. However, here we did not directly evaluate SIINFEKL cross-presentation, which is one of the limitations of the present study. Another limitation of the present study is that we could not detect platelet activation *in vitro* by here used potent adjuvant combination of anti-CD40/poly(I:C), while other studies demonstrated platelet toxicity via CD40 ligation (61, 62). However, since platelet-splenocyte interactions were enhanced in the presence of adjuvant *in vivo*, and a similar effect was observed in our co-culture experiments upon PAR4AP-treatment, we speculate that anti-CD40/poly(I:C) adjuvant induces platelet activation *in vivo*.

In addition to the effects of platelets on liposomal uptake by cDC1, we observed decreased CCR7 expression by DCs in the absence of platelets. In humans, platelets and CXCL4 have been shown to upregulate CCR7 expression in monocyte-derived DCs (63, 64). Since CCR7 is a master regulator of migration of antigen-experienced DCs towards T cell-zones, where the T cell response takes place (65, 66), a decrease in CCR7 expression may result in lower T cell activation. In addition, platelets can contribute to T cell migration by their production of CCL5 (67). CCL5 was initially described as T cell-derived chemokine that attracts and activates T cells and plays an important protective role in viral infections (68, 69). The absence of CCL5 was previously found detrimental for T cell fitness leading to T cell dysfunction and exhaustion (70). Here, CCL5 levels were significantly reduced upon platelet depletion, suggesting that platelet could support T cell immunity by promoting T cell recruitment via CCL5. Together these results indicate that in the absence of platelets, expression of chemokine receptors and chemokines are suboptimal for T cell priming.

Other reports have also described platelet-mediated T cell immunity although mainly via CD40L-mediated DC maturation (17, 26, 71). Experiments performed with bone marrow-derived and monocyte-derived DCs provide evidence for platelet-induced upregulation of maturation markers such as CD80, CD83 and CD86 and increased production of pro-inflammatory cytokines including IL-12 (17–19, 24, 72). Since platelets are the major source of CD40L in blood and CD40L is known to cross-link CD40 expressed by DCs, few studies have also addressed the impact of platelets on DC activation by scrutinizing the CD40-CD40L axis (21, 22, 25, 73). In our study platelet depletion had no effect on the expression of co-stimulatory markers by DCs. Also plasma levels of Th1-related cytokines IL-12 and IFNγ at 16h p.i. were unchanged. This is very likely due to the strong adjuvant effect of the activating CD40 antibody used in this study, which directly stimulates DC maturation.

Furthermore, we investigated the platelet binding to CD169/Siglec-1 Fc and platelet-CD169^+^ macrophage interaction. Platelets express proteins on their surface that are highly sialylated, such as GPIbα, and that can serve as a potential ligand for CD169. Our *in vitro* findings showed that platelets indeed can bind CD169/Siglec-1, however further studies are required to identify the glycoprotein or glycolipid that mediates this interaction. We also observed that PAR4AP exposure augmented platelet association with CD169/Siglec-1 Fc, which is in contrast to previous reports where platelet activation led to sialic acid removal by neuramidase (38, 74, 75). Nevertheless, we have not investigated whether incubation with PAR4AP leads to desialylation in our *in vitro* experimental set up. Next to that, PAR4 stimulation results in numerous plasma membrane events e.g. receptor exposure and release of soluble factors, that could affect Siglec-1 Fc binding to platelets.

Although controversial, antigen presenting function have been previously attributed to platelets and megakaryocytes (76–78). Platelets were described to be equipped with antigen presenting machinery, including MHCI molecules which they can express or adsorb from the environment (79). Interestingly, Zufferey and colleagues (77) provide evidence for cross-presentation in megakaryocytes and postulate that they can transfer the antigen-loaded MHC class I molecules to proplatelets thereby promoting antigen spreading. Albeit in the present study the antigen presenting capacity of platelets was not evaluated, CD8^+^ T cell responses are completely abrogated in Batf3KO animals that lack cDC1. This observation underlines the strong cDC1 dependency and thereby exclude direct activation of naïve T cells by platelets in our experimental setup (29–31, 59). However, since MHC class I molecules can be exchanged and taken up by DCs in a process called cross-dressing (80–82), we cannot exclude that cDC1 express MHC class I molecules on their surface that were originally derived from platelets.

In summary, in the present study we demonstrate that platelets act as enhancer of liposome-induced T cell immunity (**Figure S4**). While the underlying mechanism requires further investigation, our results do indicate that platelets engage in the cross-talk between liposomes, CD169^+^ macrophages and cDC1. Moreover, the observed effect could present a general mechanism for platelet contribution to adaptive immune responses after liposomal vaccination. It would be interesting to address the role of platelets in T cell induction in other vaccination strategies and/or viral infection models. Our results set the stage for further research that should fully reveal mechanism(s) underlying platelet-dependent T cell immunity in the context of liposomal vaccination. Once this is established, it will open up an avenue for interference within described networks and guide design of therapeutic strategies including vaccinations.

## Data availability statement

The raw data supporting the conclusions of this article will be made available by the authors, without undue reservation.

## Ethics statement

The animal study was approved by National Committee for Animal Experiments. The study was conducted in accordance with the local legislation and institutional requirements.

## Author contributions

JG: Conceptualization, Data curation, Formal Analysis, Investigation, Methodology, Validation, Writing – original draft, Writing – review & editing, Visualization. VL: Methodology, Resources, Writing – review & editing, Investigation. KO: Investigation, Writing – review & editing. MNT: Investigation, Writing – review & editing. AA: Investigation, Writing – review & editing. MB: Investigation, Methodology, Resources, Writing – review & editing. IJ: Resources, Methodology, Writing – review & editing, Supervision. AV: Methodology, Writing – review & editing. CK: Resources, Writing – review & editing. YvK: Supervision, Writing – review & editing, Funding acquisition. GS: Resources, Writing – review & editing, Funding acquisition. C'tV: Funding acquisition, Methodology, Resources, Writing – review & editing. JdH: Conceptualization, Funding acquisition, Supervision, Writing – original draft, Writing – review & editing.

## Supplementary material

Supplementary Figure 1 Costaining of CD41 and liposomes in macrophages and DCs *in vivo*, related to **Figure 3**. Platelet proficient mice were injected i.v. with GM3 liposomes (93 nmoles) in the presence of anti-CD40/poly(I:C) (each 25 µg/mouse) adjuvant and frequency of DiD^+^ events of CD41^+^ platelet-bound cells was determined at 16 h p.i. by flow cytometry. The data are from three independent experiments (n = 15). Each symbol represents one mouse. Error bars indicate mean ± SEM.

Supplementary Figure 2 Two step depletion of platelets. Platelets were depleted by an i.p. injection with 50 µl of polyclonal rabbit anti-mouse thrombocyte antiserum, 24 h later followed with 1,75 ug/g mouse of monoclonal anti-mouse GPIbα antibody i.v. (A) 24 h after the first injection almost complete platelet depletion was achieved (in whole blood) and this was also observed at (B) 2 h and (C) 16 h post immunization that was 24 h after the second injection (in PRP). The data are from one experiment (n=3) (A) and multiple independent experiments (B) and (C) (n = 12-25). Each symbol represents one mouse. Error bars indicate mean ± SEM. Statistical analysis unpaired T test (**p <* 0.05, ***p <* 0.01, ****p <* 0.001, *****p <* 0.0001).

Supplementary Figure 3 Platelets augment GM3 liposome uptake by F4/80^+^ macrophages and cDC1 *in vivo*, related to **Figure 3**. Platelet proficient and platelet-deficient mice were injected i.v. with GM3 liposomes (93 nmoles) in the presence or absence of anti-CD40/poly(I:C) (each 25 µg/mouse) adjuvant and DiD gMFI of GM3 liposome-bound cells was determined at 16 h p.i. by flow cytometry. GM3, GM3 liposomes; adj, adjuvant; PLT dplt, platelet-depleted. The data are from two independent experiments representative of three independent experiments (n = 15). Each symbol represents one mouse. Error bars indicate mean ± SEM. Statistical analysis one way ANOVA with Tukey’s multiple comparison test (**p <* 0.05, ***p <* 0.01, ****p <* 0.001, *****p <* 0.0001).

Supplementary Figure 4 Summary of the main findings and working hypothesis.
