# Supplementary figures and images for: Platelets interact with CD169+ macrophages and cDC1 and enhance liposome-induced CD8+ T cell responses
Source: Front Immunol. 2023 Nov 20;14:1290272. doi: 10.3389/fimmu.2023.1290272 (PMC10694434; doi:10.3389/fimmu.2023.1290272)

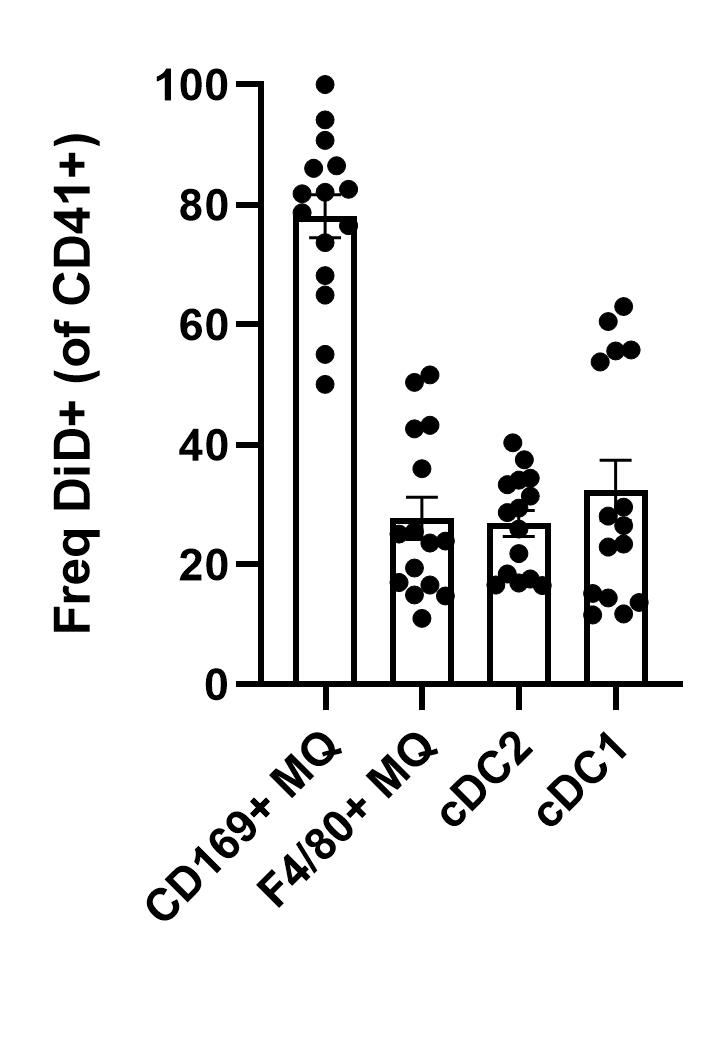

Supplement: Supplementary Figure 1 — Costaining of CD41 and liposomes in macrophages and DCs in vivo, related to Figure 3 . Platelet proficient mice were injected i.v. with GM3 liposomes (93 nmoles) in the presence of anti-CD40/poly(I:C) (each 25 µg/mouse) adjuvant and frequency of DiD+ events of CD41+ platelet-bound cells was determined at 16 h p.i. by flow cytometry. The data are from three independent experiments (n = 15). Each symbol represents one mouse. Error bars indicate mean ± SEM. [file Image_1.jpeg]

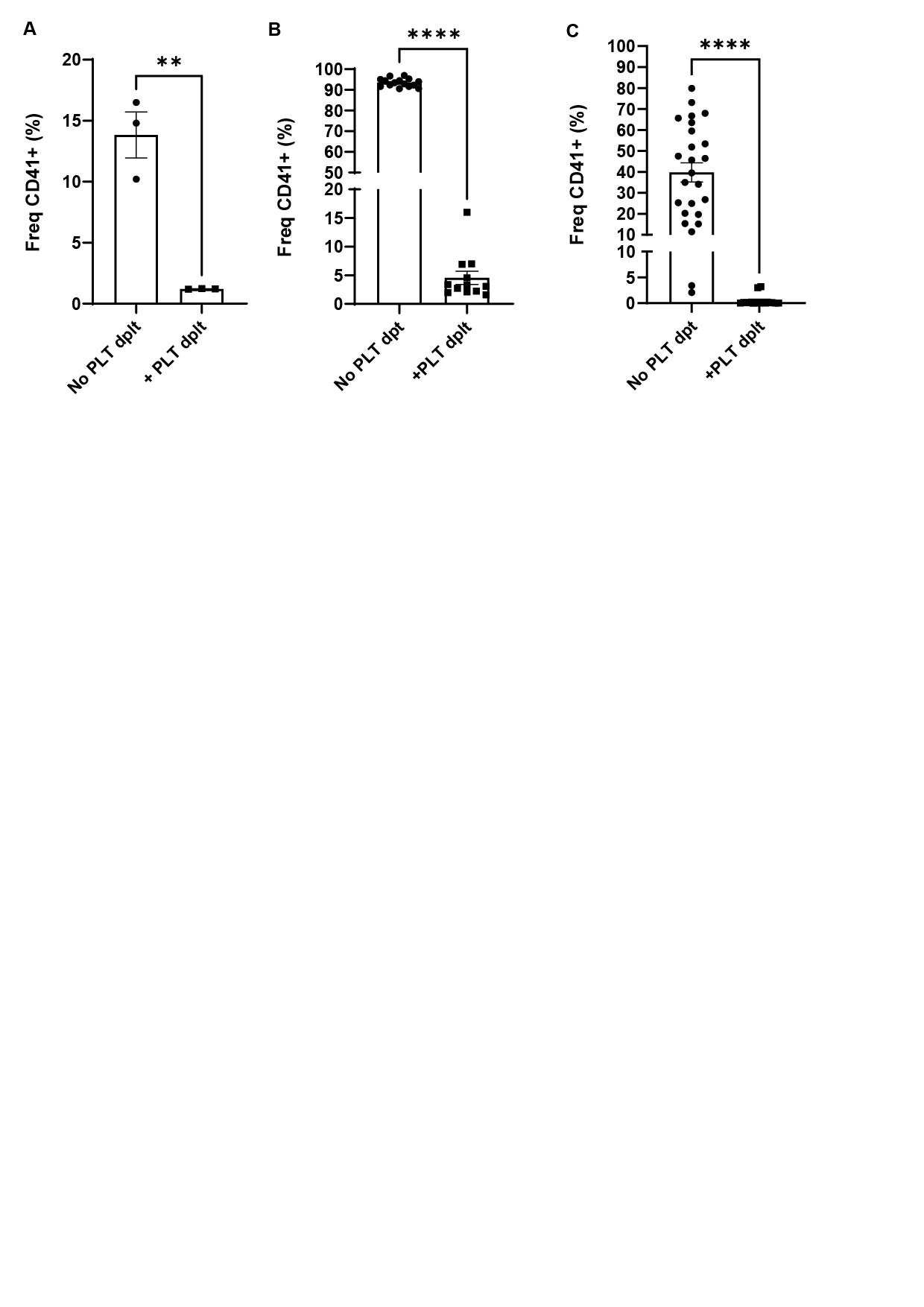

Supplement: Supplementary Figure 2 — Two step depletion of platelets. Platelets were depleted by an i.p. injection with 50 µl of polyclonal rabbit anti-mouse thrombocyte antiserum, 24 h later followed with 1,75 ug/g mouse of monoclonal anti-mouse GPIbα antibody i.v. (A) 24 h after the first injection almost complete platelet depletion was achieved (in whole blood) and this was also observed at (B) 2 h and (C) 16 h post immunization that was 24 h after the second injection (in PRP). The data are from one experiment (n=3) (A) and multiple independent experiments (B) and (C) (n = 12-25). Each symbol represents one mouse. Error bars indicate mean ± SEM. Statistical analysis unpaired T test (*p < 0.05, **p < 0.01, ***p < 0.001, ****p < 0.0001). [file Image_2.jpeg]

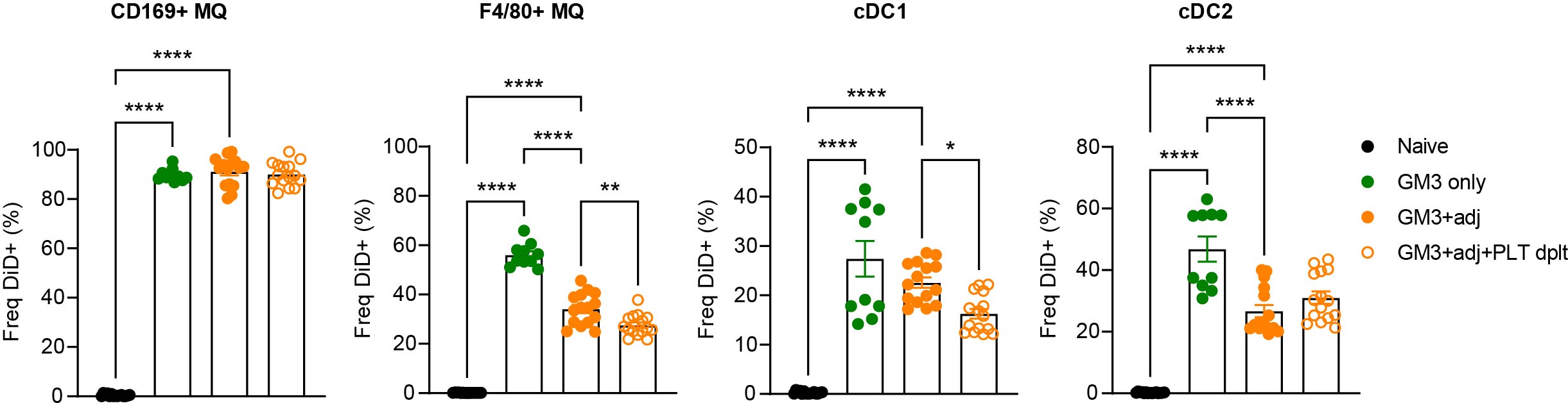

Supplement: Supplementary Figure 3 — Platelets augment GM3 liposome uptake by F4/80+ macrophages and cDC1 in vivo, related to Figure 3 . Platelet proficient and platelet-deficient mice were injected i.v. with GM3 liposomes (93 nmoles) in the presence or absence of anti-CD40/poly(I:C) (each 25 µg/mouse) adjuvant and DiD gMFI of GM3 liposome-bound cells was determined at 16 h p.i. by flow cytometry. GM3, GM3 liposomes; adj, adjuvant; PLT dplt, platelet-depleted. The data are from two independent experiments representative of three independent experiments (n = 15). Each symbol represents one mouse. Error bars indicate mean ± SEM. Statistical analysis one way ANOVA with Tukey’s multiple comparison test (*p < 0.05, **p < 0.01, ***p < 0.001, ****p < 0.0001). [file Image_3.jpeg]

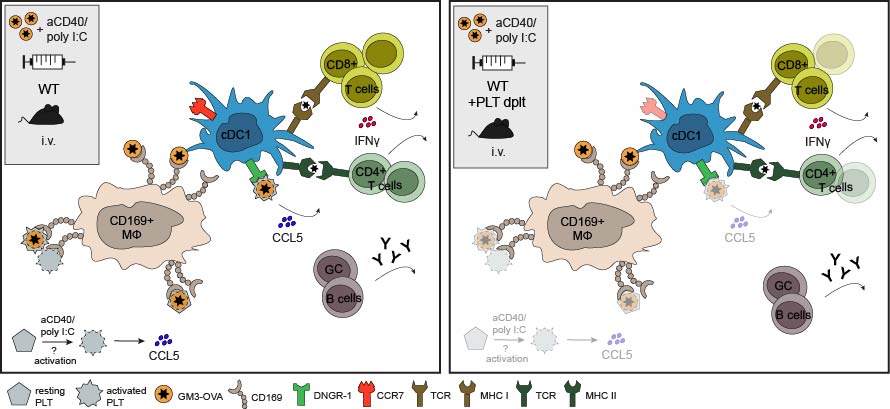

Supplement: Supplementary Figure 4 — Summary of the main findings and working hypothesis. [file Image_4.jpeg]
